# The Evaluation of Astaxanthin Effects on Differentiation of Human Adipose Derived Stem Cells into Oligodendrocyte Precursor Cells

**Published:** 2018

**Authors:** Nazem Ghasemi

**Affiliations:** Department of Anatomical Science and Molecular Biology, Faculty of Medicine, Isfahan University of Medical Sciences, Isfahan, Iran

**Keywords:** Adult stem cells, Astaxanthin, Multiple sclerosis, Oligodendroglia

## Abstract

**Background:**

Multiple Sclerosis (MS) has been explained as an autoimmune mediated disorder in central nerve system. Since conventional therapies for MS are not able to stop or reverse the destruction of nerve tissue, stem cell-based therapy has been proposed for the treatment of MS. Astaxanthin (AST) is a red fat-soluble xanthophyll with neuroprotection activity. The aim of this study was evaluation of pre-inducer function of AST on differentiation of human Adipose-Derived Stem Cells (hADSCs) into oligodendrocyte precursor cells.

**Methods:**

After stem cell isolation, culture and characterization by flow cytometry, hanging drop technique was done for embryoid body formation. In the following, hADSCs were differentiated into oligodendrocyte cells in the presence of AST at various concentrations (1, 5, and 10 *ng/ml*). Finally, immunocytochemistry and real-time PCR techniques were used for assessment of oligodendrocyte differentiation.

**Results:**

Flow cytometry results indicated that hADSCs were CD44, CD49-positive, but were negative for CD14, CD45 markers. In addition, immunocytochemistry results revealed that, in AST treated groups, the mean percentage of Olig 2 and A2B5 positive cells increased especially in 5 *ng/ml* AST treated group compared to control group (p<0.001). Moreover, real-time PCR analysis confirmed the results of immunocytochemistry.

**Conclusion:**

Since hADSCs have the potential to differentiate into multi lineage cells and due to important functions of AST in regulating various cellular processes, it seems that AST can be used as a promoter for oligodendrocyte differentiation of hADSCs for being used in cell transplantation in multiple sclerosis.

## Introduction

Multiple Sclerosis (MS) has been explained as an autoimmune-mediated disorder which is characterized by central nervous system lesions. This abnormal condition can develop a complex pattern of physical or cognitive disability as well as neurological defects^1^.

Unfortunately, the exact etiology of MS remains unclear. In spite of this, genetic predispositions, together with environmental factors such as exposure to infectious agents, vitamin D deficiencies and smoking have a major role in MS development^2–5^. Meanwhile, multifocal zones of inflammation due to immune cell infiltrations and oligodendrocyte death can trigger a cascade of events which lead to nerve demyelination^6^. As a result, the myelin sheath destruction and astrogliosis formation occurs in both white and gray matters which can interfere with correct transmission of nerve impulse and lead to neuronal dysfunction.

Since conventional therapies for MS are not able to stop or reverse the destruction of nerve tissue, stem cell-based therapy has been proposed for the treatment of neurodegenerative diseases^7,8^. In previous studies, human embryonic stem cells, human bone-marrow-derived MSCs and human placental mesenchymal stem cells were transplanted in animal models of MS. According to the result of these studies, the main mechanisms responsible for these therapeutic effects are related to neurotrophic function and differentiation potential of stem cells^7,9–10^. Other experiments demonstrated that human dental pulp stem cells and Wharton’s jelly stem cells are able to differentiate into oligodendrocyte precursor cells^11,12^. In addition, these cells are able to promote the remyelination process and significantly decrease the clinical signs of MS when transplanted in animal model of MS.

Human Adipose-Derived Stem Cells (hADSCs) are a kind of adult stem cells which have specific features including immunomodulatory and neuroprotective effects. In addition, these cells are able to differentiate into other cells outside their lineage and can be used to cell transplantation in animal models of MS^7,13^.

Despite the beneficial effects of stem cell transplantation, the serious adverse events of this manner such as tumorigenic potential should not be denied. Thus, cell transplantation using differentiated cells may decrease serious adverse complications of stem cell therapy. To this end, molecular pathways and their ligands as well as neuroprotective factors which are involved in stem cell differentiation have been studied for having access to a homogeneous population of differentiated cells.

Astaxanthin (AST) (3, 3′-dihydroxy-ß, ß′-carotene-4, 4′-dione) is a red fat-soluble xanthophyll that is found in various microorganisms^14^. This pigment in comparison to other carotenoids can prevent or reduce the risk of various human abnormalities such as neurodegenerative diseases^15,16^. AST due to special molecular structure can be connected to cell membrane and exert several biological activities than other antioxidants^17^. It has been reported that AST has significant effects on immune function both *in vitro* and *in vivo*^18,19^. For example, AST can improve immune cell proliferation and reduce humoral immune response and increase immunoglobulin production^20,21^.

According to previous published data, AST is used as a promising agent for treatment of inflammation due to protective effects against inflammation and apoptosis in epithelial cells^22^. In addition to the above mentioned effects of AST, this agent also has other effects including neuroprotection activity^23^, anti-lipid peroxidation^24^, and anticancer activity^25^. Due to the broad biological activity of AST, in this study, the pre-inducer function of AST on differentiation of hADSCs into oligodendrocyte progenitor cells was assessed.

## Materials and Methods

### Isolation and culture of hADSCs

All other chemicals, unless specified otherwise, were prepared from Sigma-Aldrich, St. Louis, MO, USA. Meanwhile, all procedures were approved by the Ethics Committee of Isfahan University of Medical Sciences (ethical code: 194267). After receiving informed consent of patients who admitted to Al-Zahra Hospital (Isfahan, Iran), hADSCs were isolated from human abdominal fat that were collected from lipoaspirate samples of three female donors (age range: 20–40 years) and cultured. According to a previous study^26^, the obtained samples were washed extensively with Phosphate-Buffer Saline (PBS) in order to remove contaminating debris and then carefully dissected and minced. Following that, the samples were enzymatically dissociated for 30 *min* at 37°*C* using 0.075% collagenase type I (Invitrogen, UK). After this period, this solution was neutralized with an equal volume of Dulbecco’s Modified Eagles Medium (DMEM/F12) (Gibco BRL, Paisley, UK) supplemented with 10% Fetal Bovine Serum (FBS) (Gibco) and then centrifuged for 10 *min* at 1200 *g*. The cellular pellet was resuspended in DMEM/F12, 10% FBS and 1% penicillin/streptomycin solution and was cultured in 25 *cm*^2^ flasks for 4–5 days until they reached approximately 90% confluency in a 37°*C* humidified incubator with a 5% CO_2_ environment.

### Cell surface marker characterization

Flow cytometric analysis was used for investigating the cell surface markers according to previous study^27^. For this purpose, 10×10^6^ hADSCs were collected from passage 3 cultures and washed twice with PBS. After removing the supernatant, the cell pellet was incubated with respective fluorochrome-conjugated antibodies against CD14, CD44, CD45 and CD90 (3 *μl*/10^6^ cells) (Chemicon, Temecula, CA, USA) for 30 *min* on ice. In addition, for isotype control, nonspecific FITC-conjugated IgG was substituted for the primary antibodies. After incubation, the cells were washed with PBS and after centrifuging (800×*g* for 5 *min*), cell pellet was resuspended in 500 *μl* of solution buffer and was transferred to flow cytometry tubes. Finally, the percentages of fluorescent cells were analyzed by a flow cytometer (Becton Dickinson, San Jose, CA).

### Hanging drop technique

For embryoid body formation, 1×10^6^ hADSCs in 15 *μl* DMEM/F12 supplemented with 2% B27, 20 *ng*/*ml* Human Epidermal Growth Factor (H-EGF), 20 *ng*/*ml* human basic Fibroblast Growth Factor (hbFGF) suspended on the inner side of tissue culture dishes were cultured for 48 *hr* under standard conditions. After this time, the embryoid bodies were collected and were split in single cells and were cultured for 4 days under standard conditions in the previous medium.

### Induction of oligodendrocyte differentiation

After cell confluency, hADSCs were dissociated using Trypsin/EDTA and seeded at 1×10^5^ *cells/cm* on 24 well TC plates (coated with 0.1 *mg/ml* poly-D-lysine) and were cultured in a pre-differentiation medium consisting of DMEM/F12, 1×NEAA, L-glutamine (2 *mM*), 1×N2, 1×B27, Shh (200 *ng/ml*), retinoic acid (2 *μM*) and different concentrations (1, 5 and 10 *ng/ml*) of AST. After 10 days, the pre-differentiation medium was replaced with DMEM/F12, 1×NEAA, L-glutamine (2 *mM*), 1×N2, 1×B27, NT3 (30 *ng/ml*), Platelet-Derived Growth Factor alpha (PDGFα) (10 *ng/ml*) and different concentrations (1, 3 and 10 *ng/ml*) of AST for 2 weeks. Meanwhile, the control group cells were differentiated in differentiation medium similar to other groups but in the absence of AST.

### Immunocytochemistry technique

In the following oligodendrocyte differentiation, differentiated cells were fixed with 4% Paraformaldehyde (PFA) for 15 *min* at room temperature, incubated in 1% BSA/10% normal goat serum/0.3 *M* glycine in 0.1% PBS-Tween for 1 *hr* to permeabilise the cells and block non-specific protein-protein interactions. In the following, incubation with primary antibodies diluted in PBS with 0.1% BSA, overnight at 4°*C* in humidified condition was done. The following antibodies were used: anti-A2B5 antibody, 1 *μg/ml*; Abcam; anti-olig2 antibody, 1:1000; Abcam. After cell washing, the slides were treated with goat anti-mouse FITC (1:500; Abcam, UK)-conjugated secondary antibodies diluted in PBS with 0.1% BSA at RT for 1 *hr*. After this time, cells were washed and fixed with 4% PFA for 5 *min* at room temperature. Finally, the nuclei were stained with DAPI for cell counting using fluorescence microscope (Olympus, BX51, Japan). For quantitative analysis, the number of A2B5, olig2 positive cells was counted on each acquired image in a minimum total of 200 cells per slide.

### Real-time PCR

In order to evaluate gene expression, total RNA was extracted from 1×10^5^ differentiated and non differentiated cells using RNeasy micro Kit (Qiagen) according to previous study^28^. After RNA dissolving in DEPC-treated water, cDNA synthesis was done using Revert-Aid First Strand cDNA Synthesis Kit (Fermentas, Germany). According to the manufacturer’s instructions, 2 *μg* of RNA was used for cDNA synthesis. Finally, quantitative real-time PCR was done using Thermal Cycler Rotor-Gene in a total volume of 20 *μl* containing Power SYBR Green master mix (2×), forward and reverse Primers (0.5 *μM*), cDNA (30 *ng/μl*) and H_2_O. At the end of this procedure, the mRNA expressions were measured for Olig2, PDGFRα (specific oligodendrocyte precursor genes), astrocyte specific marker (GFAP) and a housekeeping gene (GAPDH). Meanwhile, the sequences of all primers are presented in table 1.

**Table 1.** Primer sequences used for real-time PCR analysis

**Gene primers**	**sequence**
**Olig2**	F: 5′-CGCAGCGAGCACCTCAAATCTAA-3′ R: 5′- CCCAGGGATGATCTAAGCTCTCGAA-3′
**PDGFRα**	F: 5′- GTG GGA CAT TCA TTG CGG A-3′ Rev: 5′ AAG CTG GCA GAG GAT TAG G-3′
**GFAP**	F: 5′- CCGACAGCAGGTCCATGTG-3′ Rev: 5′-GTTGCTGGACGCCATTGC-3′
**GAPDH**	F: 5′- GAAATCCCATCACCATCTTCCAGG-3′ Rev: 5′-GAGCCCCAGCCTTCTCCATG-3′

### Statistical analysis

For data analysis, independent sample t-test and one-way analysis of variance (ANOVA) were used. Data are presented as mean±SEM and to determine the statistical significance between data, p<0.05 was considered to be statistically significant. Meanwhile, all experimental procedures were repeated at least three times.

## Results

### HADSCs and oligodendrocyte characterization

**Figure 1.**
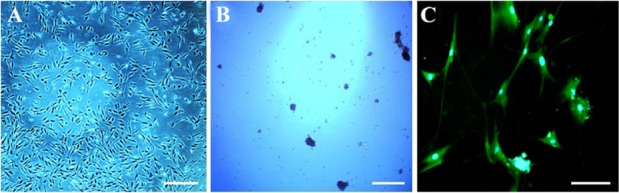
Phase contrast images of cell morphology. Morphological changes were observed in human adipose-derived stem cells (hA DSCs) during oligodendrocyte differentiation. Cultured hADSCs in passage three (A), embryoid body formation (B) and oligodendrocyte differentiation at the end of differentiation process (C). Scale bars represent 200 *μm* in A, B, and 1000 *μm* in C.

**Figure 2.**
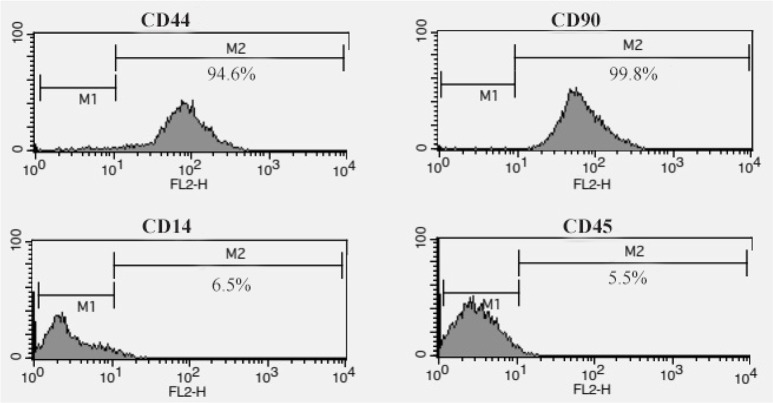
Flow cytometric analysis of hADSCs which were CD44/CD90-positive and were negative for CD14 and CD45 (hematopoietic markers).

As shown in figure 1A, hADSCs in primary culture exhibited fibroblast-like morphology. After hanging drop, the cells were aggregated and composed embryoid bodies (Figure 1B). Moreover, in the end-stage of differentiation, multipolar morphology with extensive processes was seen in differentiated cells (satellite-like morphology) (Figure 1C). In addition, flow cytometry analysis of hADSCs within passage 3 showed that hADSCs were CD44 and CD90 positive, but were negative for CD14, CD45 (hematopoietic markers) (Figure 2).

### Immunocytochemistry study of differentiated cells

**Figure 3.**
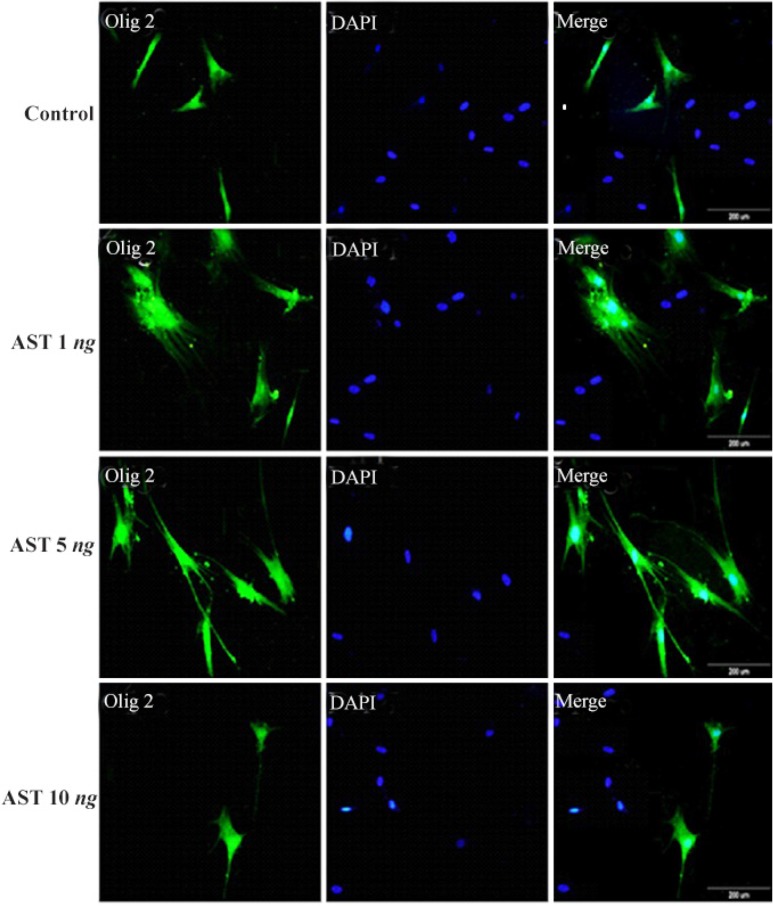
Immunostaining of differentiated cells. Differentiated cells were stained with anti Olig2. DAPI was used for nuclear counterstaining (blue) in order to show all the cells. Increased expression of Olig2 in 5 *ng/ml* astaxantin treated group was noted. Scale bar=200 *μm*

**Figure 4.**
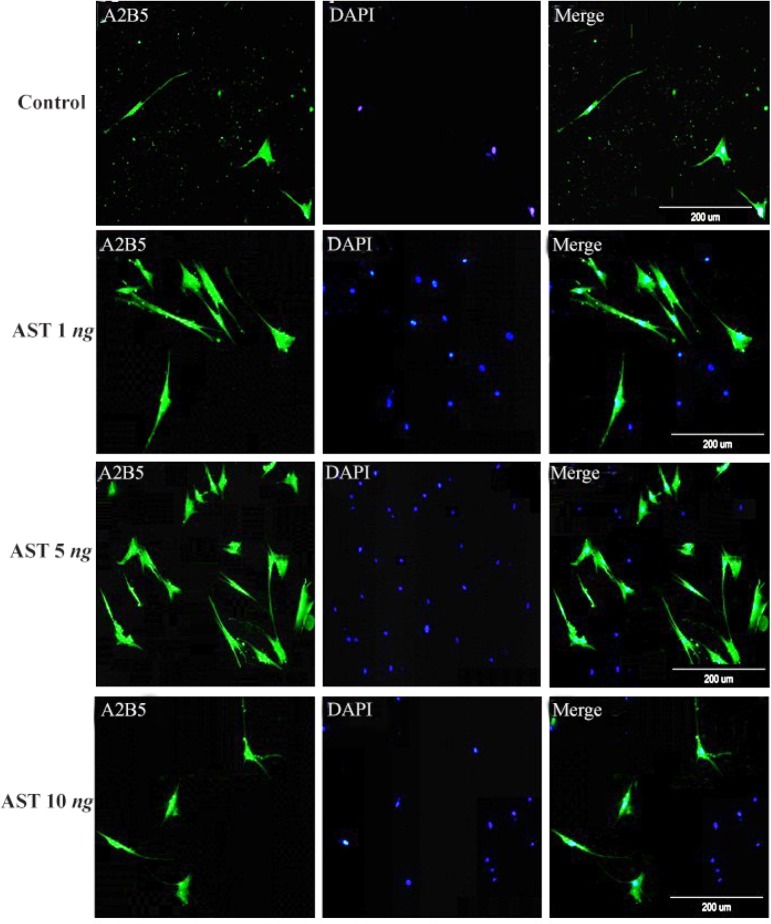
Immunostaining of differentiated cells. Differentiated cells were stained with anti A2B5. DAPI was used for nuclear counterstaining (blue) in order to show all the cells. Increased expression of A2B5 in 5 *ng/ml* astaxantin treated group was noted. Scale bar=200 *μm.*

**Figure 5.**
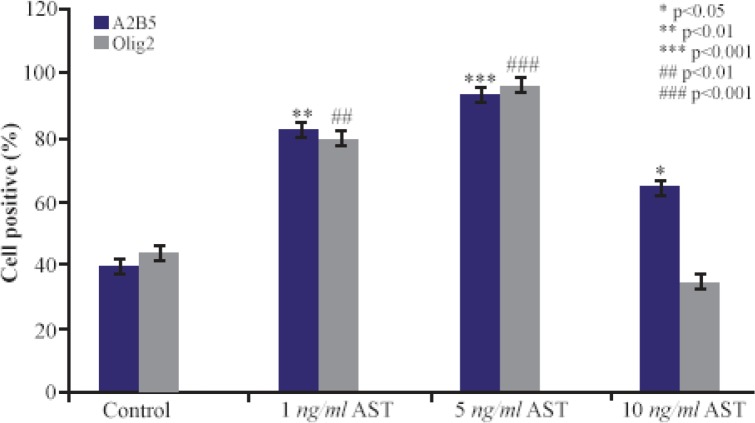
The effects of AST on A2B5 and Olig2 expression. The mean percentage of cells which expressed oligodendrocyte precursor marker was significantly increased in AST treated groups (especially in 5 *ng/ml* AST group) compared to control group.

Immunocytochemistry staining with cell type specific markers was used to identify the phenotypes of differentiated cells (Figures 3 and 4). Fluorescence microscopic analysis revealed that the mean percentage of Olig 2 and A2B5 positive cells increased in all AST treated groups especially in 5 *ng/ml* AST treated group in comparison to control group (p<0.001) (Figure 5).

### Real-time PCR results

**Figure 6.**
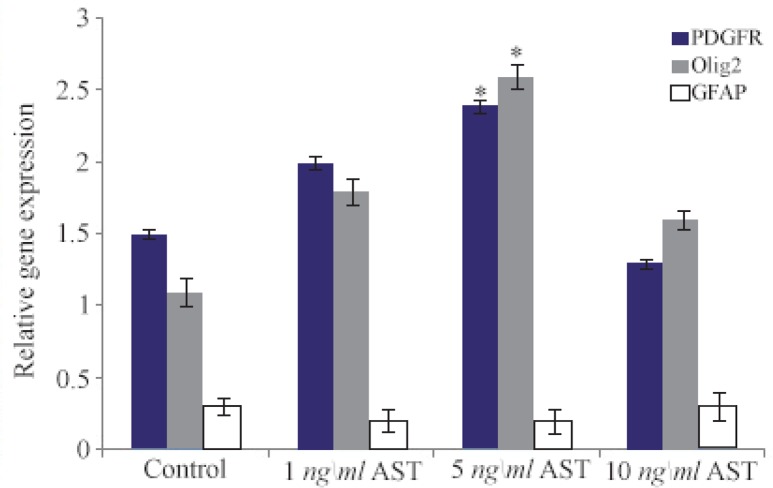
Comparative analysis of Olig2, PDGFRα and GFAP markers using real time RT-PCR. Quantification was done in oligodendrocytes after normalization to GAPDH. The expression levels of Olig2 and PDGFRα increased significantly in 5 *ng/ml* AST treated group compared to control group (p<0.05).

After RNA extraction from differentiated cells, real-time PCR assay was done. To this end, GAPDH was used as a control marker. Finally, our results revealed that the expression of Olig2 and PDGFRα markers (oligodendrocyte precursor markers) were higher in comparison to control group which is consistent with the results of immunocytochemistry (Figure 6). In addition, the GFAP expression was low which suggests that the differentiation of hADSCs into oligodendrocyte cells was favorable.

## Discussion

MS is one of the most autoimmune-mediated disorders which is usually detected in young adults. Due to progressive myelin destruction which is created during this abnormal condition, severe physical or cognitive disabilities as well as neurological problems can occur. The formation of CNS plaques as a result of focal immune cells infiltration is the primary cause of damage in MS^6^. Thus, the conventional therapy for MS is based on the use of immunosuppressive agents^29^. Since this strategy can’t suppress the MS progression, cell-based therapy has been suggested for the treatment of this pathological condition^7,8^.

Adipose Derived Stem Cells (ADSCs) due to their special characteristics such as the ability to differentiate into oligodendrocyte cells can be used for cell based therapy in MS. These cells *via* production of several growth factors such as nerve growth factor and brain-derived neurotrophic factor as well as myelin shell components^30^ have a significant role in remyelination and maintenance of the CNS functions. In our previous study, hADSCs were transplanted in lysolecithin model of MS. The results of this study revealed that hADSCs are able to differentiate into oligodendrocytes and improve remyelination process and motor functions may improve^7^.

Unlike these studies which offered the beneficial potential of stem cell therapy, the serious adverse events of this method such as tumorigenic potential cannot be denied^11^. Thus, transplantation of differentiated cell instead of stem cells may be a safe procedure. *In vitro* stem cell differentiation can be done using several promoting factors such as nerve growth factors and antioxidants. Thus, in this study, pre-inducer function of AST was assessed on differentiation of hADSCs into oligodendrocyte progenitor cells.

As shown in figure 1B, hADSCs were aggregated after hanging drop and composed embryoid bodies. Hanging drop because of providing a three dimensional micro-environmental niche for cells can facilitate the cell differentiation *via* providing direct interaction among cell population^31^.

Another relevant finding of our study was that AST is able to induce the expression of Olig2 and A2B5 markers (oligodendrocyte progenitor’s cells) in a dose-dependent manner (Figures 3 and 4). In particular, 5 *ng/ml* AST administered revealed the highest effects on hADSCs differentiation. Thus, 5 *ng/ml* AST applied was determined to be the optimal treatment for studying hADSCs differentiation into oligodendrocytes. In addition, the results of real-time PCR revealed that differentiated cells expressed oligodendrocyte precursor markers in high level which is consistent with the results of immunocytochemistry (Figure 6). Moreover, the expression of GFAP gene was in low level in all groups (especially in 5 *ng/ml* AST treated group) representing limited differentiation of hADSCs into astrocyte cells.

Previous reports have shown that AST has considerable neuroprotection activities^23^. Thus, AST is capable to trigger production of several nerve growth factors that have important role in regulation of the various cellular processes such as cell proliferation, differentiation, and maturation. As a result, AST especially in 5 *ng/ml* dosage seems to be an ideal agent for stem cell differentiation induction.

## Conclusion

The results of this study indicate that AST *via* protective effects on gene expression is able to promote the differentiation of hADSCs into oligodendrocyte progenitor cells. So, AST has beneficial therapeutic effects for cell therapy in the treatment of neurodegenerative diseases such as MS.
